# Management of Acute Promyelocytic Leukemia at Extremes of Age

**DOI:** 10.3390/cancers15143637

**Published:** 2023-07-15

**Authors:** Sabine Kayser, Shannon E. Conneely

**Affiliations:** 1 Institute of Transfusion Medicine and Immunology, Medical Faculty Mannheim, Heidelberg University, 68167 Mannheim, Germany; 2 NCT Trial Center, National Center of Tumor Diseases, German Cancer Research Center (DKFZ), 69120 Heidelberg, Germany; 3 Department of Pediatrics, Baylor College of Medicine, Texas Children’s Hospital, Houston, TX 77030, USA; shannon.conneely@bcm.edu

**Keywords:** acute promyelocytic leukemia, elderly patients, pediatric patients, treatment

## Abstract

**Simple Summary:**

This review summarizes various unique aspects of managing APL in very elderly and pediatric patients, particularly treatment with ATO, toxicities, and dose modifications.

**Abstract:**

Tailored treatment with all-trans retinoic acid (ATRA) and arsenic trioxide (ATO) has revolutionized the outcome of acute promyelocytic leukemia (APL) from a uniformly fatal disease to one of the most curable malignant diseases in humans. Due to its high efficacy, ATO/ATRA is the standard first-line therapy in younger adult, non-high-risk APL patients. However, early death is still a major issue in APL, particularly in older patients. Thus, rapid diagnostics, immediate access to ATRA-based therapy, and supportive care are of utmost importance. Nevertheless, challenging situations occur, particularly in patients excluded from controlled studies with clinical knowledge mainly based on case reports and registries. Besides the treatment of newly diagnosed patients, managing toxicities and complications remains challenging. This review discusses the approach to the treatment of APL in elderly and pediatric patients.

## 1. Introduction

Acute promyelocytic leukemia (APL) accounts for roughly 5–8% of patients with acute myeloid leukemia (AML) and is characterized by the balanced translocation t(15;17)(q22;q12), resulting in the fusion transcript *PML-RARA* [1]. Targeted therapy with all-trans retinoic acid (ATRA) has revolutionized the treatment of APL [2,3]. Single-agent ATRA can induce complete remissions (CR) in up to 80–90% of newly diagnosed and relapsed APL patients. However, CRs are often not durable [2,3,4,5,6,7]. Therefore, the standard of care in newly diagnosed APL is the combination of ATRA with chemotherapy (CTX; either an anthracycline plus cytarabine or an anthracycline alone) [8]. Recently, arsenic trioxide (ATO), in combination with ATRA, was shown to be very effective in APL [9]. Currently, ATO/ATRA is the standard therapy in adult patients with de novo, non-high-risk APL [10,11,12]. Pediatric regimens have lagged behind adult data and, thus, have been slower to incorporate ATO into the standard of care. However, regimens with ATO/ATRA are associated with a high frequency of hepatic toxicity, which normally resolves with the temporary discontinuation of ATO and/or ATRA [11]. In countries where ATO is not yet available, ATRA + CTX (e.g., ATRA + idarubicin) is still standard therapy.

Challenging situations occur in patients normally excluded from controlled studies, such as elderly and pediatric patients. Clinical knowledge of these patients is mainly based on case reports, registries, clinical trials with small sample sizes, or studies conducted with a non-randomized design. Hence, here we discuss special management considerations in these patients.

## 2. APL in the Elderly Adult

### 2.1. Epidemiology of Elderly Patients

APL seems to be rather uncommon in elderly patients [13]. However, the true incidence in this age cohort is unclear, particularly in patients beyond 70 years of age. Older patients are less likely to be transferred to an institution with expertise in hematologic conditions as comorbidities are more common in these patients, and thus APL may be under-recognized upon presentation. In addition, sudden death might be misdiagnosed as a result of a cerebrovascular accident instead of a possible APL-related cerebral hemorrhage [14]. APL accounts for a decreased proportion of AML cases as age increases (17% in patients younger than 30 years to 0.9% in patients older than 80 years) [15]. However, recent data utilizing the US National Inpatient Sample database from 2016 to 2019 suggest an increasing incidence with age, with a peak incidence of 0.62 (per 100,000 inhabitants) in patients aged 75–79 years as compared to APL’s annual age-adjusted incidence rate of 0.28 (per 100,000 inhabitants) [16]. These results were recently confirmed by a retrospective cohort analysis from the Acute Promyelocytic Leukaemia Asian Consortium Project, which included 761 newly diagnosed APL patients between 1991 and 2021 [17]. The authors observed an increasing incidence of APL in elderly (60–79 years) patients, rising from 0.22 in 1991–1999 to 0.53 in 2010–2021. The increase was >3-fold in patients older than 80, with 0.13 in 1991–1999 to 0.42 in 2010–2021. Such trends were also reported in three SEER-based (The Surveillance, Epidemiology, and End Results Program) studies covering the time periods 1992–2001 [18], 1975–2008 [19], and 2000–2014 [20].

Taken together, the incidence of APL in elderly patients is increasing, though this data may be confounded by improved recognition of APL and transfer to dedicated hematology centers over the years.

### 2.2. Risk Category

Published data in elderly patients vary with respect to the distribution of risk category according to white blood cell count (WBC) at diagnosis [21,22]. In one study, 20% (*n* = 21/104) of elderly patients were reported to have high-risk APL [21]. However, another publication reported a higher prevalence of high-risk APL, occurring in 31% (*n* = 28/91) of elderly patients [22]. Considering current outcomes with ATO/ATRA-based therapy are comparable, risk stratification based on presenting WBC may no longer be necessary [23]. 

### 2.3. Treatment and Outcome of Elderly Patients

#### 2.3.1. ATRA/CTX-Based Approach

In a previous study of 104 elderly patients (median age, 68 years; range, 60–83 years), ATRA plus an anthracycline was given successfully for both induction and consolidation therapy in APL [21]. Patients who were MRD-negative after consolidation received 2-year maintenance with 6-mercaptopurine (50 mg/m^2^/day), intramuscular methotrexate (15 mg/m^2^/week), and ATRA (45 mg/m^2^/day for 15 days every 3 months). Overall, outcomes were favorable, with an early death (ED) rate of 15%, a CR rate of 84%, a 6-year cumulative incidence of relapse (CIR) of 8.5%, and disease-free survival (DFS) of 79% [21]. Unfortunately, patients older than 70 years had a lower CR rate (74%) as compared to patients aged 60–70 years (89%) [21]. Similar results were recently reported by Martinez-Cuadrón et al. [24]. These authors compared the long-term outcome of older patients (median age, 67 years) with de novo APL treated in the LPA2005 and LPA96&99 trials [24]. Treatment in the LPA2005 trial, an age- and risk-adapted protocol with reduced post-consolidation CTX, resulted in a higher 5-year DFS (87% vs. 69%; *p* = 0.04) and 5-year OS (74% vs. 60%; *p* = 0.06) as compared to the LPA96&99 trials [24].

#### 2.3.2. Treatment-Related Mortality for ATRA/CTX-Based Therapy

Elderly patients may suffer from higher treatment-related mortality due to a higher vulnerability to treatment toxicities. In a population-based report from the Swedish Adult Acute Leukemia Registry, a high ED rate after ATRA ± anthracycline-based induction therapy (60% vs. 19%) was observed in patients above the age of 80 years as compared to patients aged 50–59 years [15]. The ED rate was associated with poor performance status, explaining the high rate in very elderly patients [15]. In a recently published retrospective analysis of 288 patients with newly diagnosed APL treated with ATRA-based induction regimens, older age (≥60 years) was the only independent risk factor for ED (hazard ratio [HR] = 15.057; *p* = 0.004) [25]. 

Sanz et al. noted that 6 of 25 (24%) patients ≥70 years died in remission [21], while Ades et al. reported that 19% of patients ≥60 years died due to complications of myelosuppression during consolidation with daunorubicin/cytarabine [26]. The high treatment-related mortality rate seems to justify a CTX-free treatment approach with ATO/ATRA in this highly vulnerable group of patients. 

#### 2.3.3. ATO/ATRA-Based Approach

Data are scarce in regards to outcome after ATO/ATRA therapy in elderly patients. In the pivotal APL0406 trial, the upper age limit was 70 years, and a few patients above 60 years were included [10]. Nevertheless, there is no biological evidence to suggest that non-high-risk APL behaves differently according to age. Thus, disease response to ATO/ATRA therapy should be similar between the elderly and younger adults. Zhang et al. reported on 33 elderly patients with de novo APL (median age, 65 years; range, 60–79 years) treated with ATO monotherapy for induction and consolidation therapy [27]. Of those, 88% achieved a CR. The 10-year CIR, OS, and DFS rates were 10.3%, 69.3%, and 64.8%, respectively. Overall, ATO was well tolerated, and all non-hematological adverse events were all manageable and reversible. Leukocytosis occurred in 64% of the patients. The non-relapse mortality (NRM) of 6.9% due to noninfectious diseases was better than [27] the 10–18.6% observed after CTX in older patients, despite reduced CTX intensities. Moreover, the NRM after CTX in elderly patients was mainly caused by infections, again arguing against a CTX-based approach [26,28,29]. None of the patients treated with ATO developed a secondary malignancy. Only one patient died of liver cancer 117 months after the achievement of CR. This patient had a longstanding hepatitis B virus infection and hepatic cirrhosis [27]. In addition, a large cohort of 433 elderly patients (median age, 73.4 years) treated either with CTX/ATRA (n = 259) or ATO-based therapy (ATO/ATRA, n = 26; CTX/ATRA+ATO, n = 148) was evaluated [23]. Response after ATO-based therapy was high, with a CR rate of 92% as compared to 82% after CTX/ATRA. Further, induction death rates were 8% and 18%, respectively. Therapy with ATO/ATRA ± CTX resulted in a significantly lower CIR as compared to CTX/ATRA (*p* < 0.001) [23]. Treatment with ATO/ATRA ± CTX was also effective in patients with high-risk APL (WBC > 10 × 10^9^/L), whereas treatment with CTX/ATRA resulted in a significantly higher CIR in high-risk as compared to low-risk patients (*p* < 0.001). Data on the development of secondary neoplasms were available in 163 patients. Of those, 11 (7%) patients developed a secondary neoplasm after a median of 61.5 months (range, 5.5–85 months) from the time point of CR. All of them were treated with CTX/ATRA. In a competing risk analysis among patients in CR after intensive therapy, the rate of secondary malignancies was significantly lower after ATO/ATRA ± CTX as compared to CTX/ATRA (*p*  =  0.02). 

The favorable results after treatment with ATO/ATRA were recently confirmed in a small cohort analysis in elderly patients by Rosati et al. [30]. Particularly, sub-optimal induction treatment with modified or reduced personalized approaches (such as ATRA monotherapy, adapted AIDA with reduced IDA to 5 mg/m^2^ or reduced number of IDA doses, ATRA+ gemtuzumab ozogamicin) led to dismal outcomes in almost half of the patients [30]. 

Thus, it seems reasonable to offer ATO/ATRA ± CTX as first-line treatment to older patients irrespective of the risk group. The application of a geriatric assessment might help in the selection of patients suitable for ATO-based therapy [31]. Table 1 gives an overview of the treatment schedule and ATO/ATRA treatment dosages according to the APL 0406 trial [10,11].

#### 2.3.4. QT Prolongation Associated with ATO

Treatment with ATO is associated with electrolyte abnormalities, thus leading to prolongation of the QT interval corrected for the heart rate (QTc). As a consequence, QTc-prolongation can lead to ventricular tachycardia and death [32,33]. Within the APL0406 trial, QTc-prolongation occurred in 12 of 77 (16%) patients after treatment with ATO/ATRA but was severe (QTc ≥ 501 msec) in only one patient. In this patient, ATO was permanently discontinued, and the patient went off protocol. There were no cases of life-threatening cardiac arrhythmias [11]. Thus, during treatment with ATO, close monitoring of the electrocardiogram (ECG) and electrolytes at least twice weekly is necessary. For ECG surveillance of QT interval prolongation, the formula by Fridericia should be used. Electrolytes should always be within the upper-normal range, particularly magnesium and potassium. Concomitant therapy QTc prolonging medications should be discontinued. Patients with episodes of significant QT prolongation, torsades de pointes, or other risk factors should be closely monitored [34]. ATO should be discontinued in patients with an absolute QTc interval >500 msec, and electrolytes should be repleted. Normalization of the QTc interval may take several days after discontinuation. ATO may be resumed as soon as the QTc is normalized.

#### 2.3.5. Dose Modifications

In the case of grade 3/4 non-hematological toxicities during treatment with ATO/ATRA, dose modifications, according to Table 2, are recommended [10,11].

#### 2.3.6. Treatment Approach

We recommend the following approach:

- Prednisone 0.5 mg/kg/day p.o. should be administered from day 1 of ATO until the end of induction therapy for differentiation syndrome prophylaxis;
- For WBC count > 10 × 10^9^/L, start hydroxyurea;
- Bone marrow evaluation on day 28;
- Induction therapy should be terminated on the basis of morphological criteria (if CR or CRi is reached on day 28);
- ATO/ATRA should be continued up to a maximum of 60 days in case CR or CRi is not achieved by day 28;
- Cytogenetic and molecular assessment at the end of induction therapy is not necessary for CR. Molecular analysis should be performed after consolidation only.

#### 2.3.7. Long-Term Toxicities with ATO/ATRA

ATO/ATRA is assumed to reduce the long-term toxicities of anthracycline therapy. However, potential long-term complications after therapy with ATO/ATRA may exist. A long-term follow-up study assessed 265 newly diagnosed APL patients who were treated with ATO/ATRA between 2001 and 2012 [35]. The median follow-up in this study was 83 months. The authors reported higher rates of grade 1 liver dysfunction (15% vs. 2%) and hepatic steatosis (43% vs. 18%) as compared to healthy controls [35]. One patient developed breast cancer three years after the termination of ATO. Eight patients developed skin lesions (hyper- or hypopigmentation or hyperkeratosis/hyperplasia) during maintenance therapy or within 6 months after treatment. All patients recovered within 2 to 18 months [35]. Common signs of chronic arseniasis, such as cardiovascular events, chronic renal insufficiency, diabetes, or neurological dysfunction, were not observed [35].

In addition, peripheral neuropathy can also be seen after treatment with ATO [36,37]. Usually, symptoms are mild and reversible after ATO discontinuation [11] but may be severe and irreversible in patients with coexisting thiamine deficiency [36].

In addition, there is a high frequency of varicella zoster virus (VZV) reactivation after ATO-based treatment. VZV reactivation occurred in 7 (46.7%) of 15 patients treated with ATO-based therapy, compared to 0 out of 10 patients treated with conventional CTX [38]. However, treatment with acyclovir or valacyclovir was highly effective, and postherpetic neuralgia was not reported. Therefore, acyclovir or valacyclovir should be given prophylactically throughout ATO-based therapy [38]. 

Recently, Norsworthy et al. reported data from 124 adult APL patients from the National Cancer Institute SEER program who were diagnosed with APL between 2006 and 2015 and received systemic APL therapy [39]. The authors performed an exploratory population-based analysis of secondary malignancies in patients treated with (*n* = 64) or without (*n* = 60) ATO. The authors concluded that there was a trend towards increased secondary malignancies after ATO-based treatment versus non-ATO-containing APL regimens (9.9% vs. 6.0% at 24 months, *p* = 0.24), although the risk was not significantly increased compared with patients receiving other APL therapies. Despite that, survival outcomes appeared better after ATO-based therapy [39], which is consistent with data from the APL 0406 trial [10,11] as well as data in elderly patients [23,30].

Larger data sets are needed to draw firm conclusions regarding the occurrence of comorbidities and organ toxicities. We suggest routine follow-up for patients after completion of APL therapy to monitor for cardiovascular risk factors as well as age-appropriate cancer screening. 

## 3. APL in the Pediatric Patient

### 3.1. Epidemiology in Children

APL is a rare form of AML in children less than 18 years of age. It accounts for less than 2% of AML in children less than 2 years old, 5–6% in children between the ages of 2 and 12, and 10–15% of children between the age of 12 and 18 years [40]. Overall, this corresponds to less than 0.1 cases per 100,000 persons less than 18 years of age. As with many pediatric malignancies, no clear risk factors for the development of APL have been identified. Laurie et al. recently published data suggesting obesity may be associated with the development of APL and influence APL-related outcomes in children, similar to that previously published in adults [41,42]. They showed that the prevalence of obesity among children with APL was 34–35% compared to population obesity rates of 20–24% during the same time period and age range. In addition, obese children with APL had inferior EFS and OS in a recent clinical trial, but obesity was not associated with an increased incidence of adverse events [41]. 

### 3.2. Clinical Features of Pediatric Patients

#### 3.2.1. Risk Category

Data regarding risk category assignment in children based on WBC at diagnosis come predominantly from clinical trials, which may not accurately represent the full spectrum of disease, as children that are very ill at presentation are less likely to be offered clinical trial enrollment. In the most recent Children’s Oncology Group Clinical Trial (NCT02339740), 63.6% of participants were categorized as standard-risk and 36.4% as high-risk. There was no difference in median age between the standard- and high-risk groups [43]. These patterns have been maintained across multiple clinical trials and are comparable to risk breakdown in adult studies [44,45]. Thus, children do not appear to be more likely to have a high or standard risk of disease compared to adults.

#### 3.2.2. Severe Coagulopathy at Diagnosis

Coagulopathy is a known feature of APL, with laboratory findings similar to disseminated intravascular coagulation. The severity can range from isolated lab abnormalities to severe bleeding, including intracranial or pulmonary hemorrhage, which can be lethal. Severe bleeding events have been reported in up to 15% of children with APL, leading to early death in up to 10% [43,44,45]. In particular, central nervous system (CNS) haemorrhage can be challenging to diagnose in young children who are unable to communicate symptoms early in the disease process. Although routine CNS imaging is not recommended to screen for CNS hemorrhage, even in nonverbal patients, many practitioners will perform head imaging at diagnosis in young children or have a lower threshold to obtain head imaging with any unexplained clinical phenomenon. Bleeding complications are seen more commonly in children with high-risk diseases, and thus extra vigilance should be taken in monitoring these patients [46,47]. 

### 3.3. Treatment and Outcome of Pediatric Patients

Given the rarity of APL in the pediatric population, clinical trials to establish the best treatment approaches are informed by advances first made in adults with APL. Due to the limited number of children with APL, these studies do not include randomization and rely on historical controls to demonstrate improved outcomes. In addition, they must be conducted by large, international groups, such as the Children’s Oncology Group and the International Consortium for Childhood APL. Thus, treatment strategies in children generally reflect those in adults but with a significant lag in the implementation of new therapies. However, the treatment of children with APL and other cancers can be complicated by family dynamics and resources as well as the limitations of their treating cancer center. As pediatric APL therapy is typically only administered at large, tertiary hospitals, patients outside of metropolitan areas may have difficulty accessing care. This is particularly true of ATO/ATRA-based therapy, where ATO is given daily during induction and 5 days per week during consolidation.

#### 3.3.1. CTX/ATRA-Based Approach

The current standard of care for children with APL utilizes a backbone of ATRA and ATO with only a limited role for conventional CTX. Previous regimens included the anthracycline idarubicin during induction therapy for all patients regardless of risk group [45]. This was followed by consolidation cycles consisting of combinations of ATRA, idarubicin, mitoxantrone, and high-dose cytarabine. All patients subsequently received maintenance CTX consisting of repeating cycles of ATRA, methotrexate, and mercaptopurine. The ICC-APL-01 trial, which enrolled children from eight different cooperative groups, was the largest study to use this approach and enrolled 258 patients under the age of 18 [48]. Although this strategy achieved overall excellent outcomes with a 5-year OS of 94.6% and EFS of 79.9%, the CIR was 14.3% at 5 years, and 3% still suffered ED due to intracranial hemorrhage. In the Children’s Oncology Group AAML0631 trial, the first consolidation cycle was replaced with an ATO/ATRA cycle in order to decrease cumulative anthracycline exposure [45]. This regimen led to 81% of patients achieving a hematologic CR at the end of induction and 100% molecular remission by the end of consolidation. The overall 3-year OS was 94%, and EFS was 91%. However, a significant difference in outcomes between standard-risk (EFS 95%; OS 98%) and high-risk (EFS 83%; OS 86%) patients was evident. DFS was more comparable at 93% for standard-risk patients and 89% for high-risk patients, and the relapse rate was only 4% across the entire cohort. All patients who suffered ED in induction were high-risk, likely contributing to the inferior outcomes in the high-risk patient cohort. In each trial, anthracycline exposure remained above 300 mg/m^2^.

Fortunately, pre-existing comorbidities that would prevent patients from receiving conventional CTX are rare in children. However, regimens that include significant amounts of anthracycline and other chemotherapeutic agents carry a significant risk of toxicity, particularly during anthracycline-containing cycles (Table 3). One study reported a 30–40% incidence of febrile neutropenia, with over 30% of patients having a documented infection during induction chemotherapy alone [45]. Another study reported 60% of children suffering febrile neutropenia during intense chemotherapy regimens [45]. Prolonged QT_c_ was seen in 17.2% of patients during ATO-containing cycles. Elevated liver enzymes were found in 21.1% of patients during maintenance chemotherapy. The incidence of non-QT_c_-related cardiac toxicities was rare. 

#### 3.3.2. ATO/ATRA-Based Approach

As adult regimens incorporated more ATO and reduced conventional CTX with outstanding results, pediatric regimens have subsequently aimed to decrease CTX-related toxicities by minimizing exposure to anthracyclines while utilizing more ATO. The most recent clinical trial led by the Children’s Oncology Group studied combination therapy with ATRA and ATO for induction and consolidation. The only difference between treatment for standard- and high-risk patients was that the latter received four doses of idarubicin during induction. All patients achieved a hematologic CR with a median time to CR of 47 days from the start of therapy [43]. Remarkably, the 2-year OS in this study was 99% for standard-risk patients and 100% for high-risk patients. Two-year EFS was 98% for standard-risk and 96.4% for high-risk patients. Three relapses occurred during this trial, all of which were salvaged with CTX, followed by autologous stem cell transplantation. An additional clinical trial from the Chinese Children’s Leukemia Group used a similar strategy with a backbone of ATO/ATRA, although they administered additional anthracycline during consolidation therapy [49]. They achieved similar results as above. Importantly, this trial allowed for the use of either RIF, an oral arsenic formulation, or the traditional intravenous formulation and showed sustained efficacy regardless of the mode of administration. Other studies investigating the use of oral arsenic have also shown sustained excellent outcomes compared to intravenous arsenic use [50]. 

One important difference between pediatric and adult ATRA-based regimens is the dose used for ATRA therapy. Clinical trials in children have traditionally used low-dose ATRA at 25 mg/m^2^/day divided into 2–3 doses. Although this dose had been used in some adult regimens in combination with conventional chemotherapy, studies suggested inferior outcomes with an increased risk of relapse at the lower dose [51]. As the rate of relapse in children treated with newer therapies is incredibly low, pediatric regimens continue to use low-dose ATRA (Table 1).

##### Toxicities

The use of ATO/ATRA-based regimens leads to a distinct side effect profile different from those seen with conventional CTX. Differentiation syndrome occurs in 25–30% of patients, with a higher incidence in children with high-risk diseases [43,45]. Grade 2 or higher QT_c_ prolongation occurs in 10–26% of patients, with the highest incidence during induction therapy, while the incidence of non-QT-related cardiac toxicities was rare [43]. In contrast to chemotherapy-based regimens, less than 10% of patients experienced febrile neutropenia during any phase of therapy. The most common non-cardiac side effects included mucositis (exclusively in high-risk patients during induction), hyperglycemia, and elevated liver enzymes. 

##### Long-Term Toxicities

Data regarding long-term complications in survivors of pediatric APL is limited due to both the rarity of the disease and the relatively recent focus on long-term survivorship by various cancer consortiums in children. There is no comprehensive data published to date detailing late toxicities in either those exposed to prior CTX-containing regimens or the newer ATO/ATRA-based therapy. In the CTX-based AAML0631 Children’s Oncology Group regimen, there were two cases of secondary malignancies: one skin cancer and one AML [45]. As arsenic exposure in the environment has been linked to skin cancer, this may have been a secondary effect of ATO therapy during this trial. Similarly, the secondary AML was likely related to anthracycline exposure and was diagnosed 40 months after diagnosis. Secondary AML was also reported in one patient in the ICC-APL-01 trial [48]. A retrospective, single-institution analysis of 67 patients treated from 2002 to 2018 did not show any chronic toxicity such as liver or renal dysfunction, hyperpigmentation, cardiac events, or secondary malignancies, with a median follow-up of 7.7 years [52]. Thus, more data is needed to accurately understand the long-term risk associated with current therapies, as many survivors of pediatric APL will go on to live many more decades.

## 4. Conclusions

APL is predominantly a disease of young adults and occurs at low frequency in pediatric and elderly populations. Current data, however, suggest that the incidence of APL in elderly patients is rising. Patients at extreme ages pose unique management challenges, predominantly due to less robust clinical trial data to guide treatment, but ones that can be overcome to provide excellent outcomes. ATO/ATRA is a feasible treatment and leads to better outcomes as compared to CTX/ATRA in the primary management of older patients with APL. Similarly, ATO/ATRA therapy in children leads to outstanding survival outcomes with lower toxicity profiles than CTX/ATRA, particularly in regard to infection risk. Age limits for the use of ATO are not included in either the US Food and Drug Administration or the European Medicine Agency. Thus, ATO/ATRA should be considered the preferred treatment in older adults with APL, irrespective of risk group, unless underlying morbidities or toxicities, such as liver dysfunction or peripheral neuropathy, preclude its safe use. In children, ATO/ATRA has been used successfully in standard-risk APL, though no trials have been conducted that fully remove CTX from induction regimens for high-risk patients. This has led to a significant reduction in morbidity and mortality associated with CTX/ATRA-based therapy. As children do not often have multiple underlying comorbidities, ATO/ATRA should be a safe option for a vast majority of patients. 

The primary drawback of ATO therapy is the need for daily infusions, which typically occur at a dedicated cancer center in metropolitan areas. This limits the ease and availability of therapy for those in rural areas, particularly in children where cancer centers are less numerous. The use of oral arsenic, currently under investigation, will further aid in optimizing care for APL patients, particularly those at extreme ages. Although ATO/ATRA therapy still carries the risk of long-term toxicities, which has been better described in adult populations, the benefits outweigh the risk except for in a few individual circumstances.

In conclusion, ATO/ATRA has been well-studied in younger adults with APL, and current data supports expanding this method to all age groups, including the elderly and pediatric populations. While this treatment likely carries long-term risks that are not yet fully understood, toxicity profiles are overall superior to CTX/ATRA-based regimens, which are associated with high rates of infection and increased risk of cardiac toxicity and secondary neoplasms. Early death remains a challenge and may require special vigilance in vulnerable patient populations.

## Figures and Tables

**Table 1:** Treatment schedule and dosages of arsenic trioxide and all-trans retinoic acid as first-line therapy in non-high-risk acute promyelocytic leukemia.

Induction			
**Drug**	Dose	Route	Administration
**ATO**	0.15 mg/kg	i.v.	over 2 h daily starting on day 1, until CR, maximally 60 days
**ATRA**	Adults: 45 mg/m^2^ Children: 25 mg/m^2^	orally	in 2 single doses daily starting on day 1, until CR, maximally 60 days; doses will be rounded up to the next 10 mg increment
**Consolidation**			
**ATO**	0.15 mg/kg	i.v.	over 2 h daily for 5 days/week, treatment break on days 6 and 7 a total of 4 weeks on and 4 weeks off for a total of 4 courses; last cycles will be administered on weeks 25–28
**ATRA**	Adults: 45 mg/m^2^ Children: 25 mg/m^2^	orally	in two single doses daily, 14 days on, 14 days off for a total of 7 courses; doses will be rounded up to the next 10 mg increment

Abbreviations: ATO, arsenic trioxide; ATRA, all-trans retinoic acid; i.v., intravenously.

**Table 2:** Dose modifications for common non-hematological toxicities.

Dose Level	0 (Start Level)	−1	−2	−3
ATO (mg/kg)	0.15	0.11	0.10	0.075
ATRA (mg/m^2^)	45	37.5	25	20

Abbreviations: ATO, arsenic trioxide, ATRA, all-trans retinoic acid.

**Table 3:** Common toxicities reported on pediatric APL clinical trials.

	COG AAML0631 ^	ICC-APL-01	COG AAML1331
Febrile Neutropenia (%)	31.4–40.9	42–60	NR
Mucositis (%)	13.7	NR	19.6
Infections (%)	33.3	11–33	NR
Elevated Liver Enzymes (%)	21.11 *	NR	1.8–15.3
Prolonged QT_c_ (%)	11.8–17.2	NR	25–58.9
Pseudotumor cerebri (%)	<6	NR	7.8

^ Values only listed for cycles where toxicity occurred in >10% of patients; * maintenance cycle only. Abbreviations: NR, not reported.
